# Analysis of the gut microbiota in children with gastroesophageal reflux disease using metagenomics and metabolomics

**DOI:** 10.3389/fcimb.2023.1267192

**Published:** 2023-10-13

**Authors:** Xiaolin Ye, Feihong Yu, Jin Zhou, Chunna Zhao, Jie Wu, Xin Ni

**Affiliations:** ^1^ Department of Gastroenterology, Beijing Children’s Hospital, Capital Medical University, National Center for Children’s Health, Beijing, China; ^2^ National Center for Pediatric Cancer Surveillance, Beijing Children’s Hospital, Capital Medical University, National Center for Children’s Health, Beijing, China

**Keywords:** pediatrics, gastroesophageal reflux disease, gut microbiota, metagenomics, metabolomics

## Abstract

**Background:**

There is no direct evidence of gut microbiota disturbance in children with gastroesophageal reflux disease (GERD). This study aimed to provide direct evidence and a comprehensive understanding of gut microbiota disturbance in children with GERD through combined metagenomic and metabolomic analysis.

**Methods:**

30 children with GERD and 30 healthy controls (HCs) were continuously enrolled, and the demographic and clinical characteristics of the subjects were collected. First, 16S rRNA sequencing was used to evaluate differences in the gut microbiota between children with GERD and HC group, and 10 children with GERD and 10 children in the HC group were selected for metagenomic analysis. Nontargeted metabolomic analysis was performed using liquid chromatography/mass spectrometry (LC/MS), and metagenomic and metabolomic data were analyzed together.

**Results:**

There were significant differences in the gut microbiota diversity and composition between children with GERD and HCs. The dominant bacteria in children with GERD were Proteobacteria and Bacteroidota. At the species level, the top three core bacterial groups were *Bacteroides stercoris*, *Bacteroides vulgatus* and *Alistipes putredinis*. The main differential pathways were identified to be related to energy, amino acid, vitamin, carbohydrate and lipid metabolism. LC/MS detected 288 different metabolites in the positive and negative ion modes between children with GERD and HCs, which were mainly involved in arachidonic acid (AA), tyrosine, glutathione and caffeine metabolism.

**Conclusion:**

This study provides new evidence of the pathogenesis of GERD. There are significant differences in the gut microbiota, metabolites and metabolic pathways between HCs and children with GERD, and the differences in metabolites are related to specific changes in bacterial abundance. In the future, GERD may be treated by targeting specific bacteria related to AA metabolism.

## 1 Introduction

Gastroesophageal reflux disease (GERD) refers to a disease in which the contents of the stomach reflux into the esophagus, causing corresponding esophageal symptoms and/or complications. The typical symptoms are heartburn, reflux, and chest pain. It can also cause extraesophageal symptoms such as chronic cough, asthma, aspiration pneumonia, and pharyngitis, which seriously affect the quality of life of patients. The prevalence of GERD ranges from 10%-20% in Western countries and 5.2%-18.3% in Asia (Jung, 2011). According to the endoscopic manifestations, it is divided into three types: nonerosive reflux disease (NERD), reflux esophagitis (RE) and Barrett’s esophagus (BE).

The traditional view is that the pathological changes involved in GERD are mainly caused by chemical damage from gastric acid or duodenal bile reflux stimulating the esophageal mucosa. As the disease progresses, the lesions gradually involve the mucosal layer, submucosal layer, muscular layer and serous layer. However, most GERD patients show no mucosal damage under endoscopy, suggesting that other pathogenic processes may be involved (Boeckxstaens et al., 2014; D'Souza et al., 2021; Sharma and Yadlapati, 2021). Recent studies have shown that interactions between the gut microbiota and the host immune system play a key role in the pathogenesis of gastrointestinal diseases, including esophago-related diseases (Gorkiewicz and Moschen, 2018; Dicks, 2022). The diversity, stability, and resilience of the gut microbiota and its responsiveness to physiological, pathological, and environmental changes make it and related metabolic pathways useful biomarkers, diagnostic tools, or therapeutic targets for numerous diseases (Magnusdottir et al., 2017; von Frieling et al., 2018).

Although some progress has been made in the study of the microbiota of GERD patients, most previous studies have focused on the microorganisms in the esophagus and stomach. Studies have shown that the esophageal microbiota of normal people mainly comprises gram-positive *Streptococcus* in Firmicutes (Hunt and Yaghoobi, 2017; Deshpande et al., 2018). However, the esophagus of GERD patients was found to be dominated by gram-negative anaerobic bacteria and trace aerobic bacteria in Bacteroidetes, Proteobacteria and Fusobacteria (Yang et al., 2009; Yu et al., 2019). In addition, chronic inflammation has been shown to play a role in the development of various gastrointestinal diseases (such as BE and esophageal cancer), and changes in the gut microbiota induced by chronic inflammation further accelerate the occurrence and development of diseases(Ghoshal et al., 2012). Lipopolysaccharide (LPS), an important component of the outer membrane of gram-negative bacteria, may promote tissue inflammation by inducing the expression of NF-κB. In animal models, a high-fat diet promoted the progression of BE to esophageal cancer by regulating the intestinal flora to upregulate the IL-8/CXCL1 inflammatory signaling pathway(Munch et al., 2019), confirming the role of the gut microbiota in disease progression. The composition and function of the gut microbiota in GERD patients remain largely unknown, and it has been shown that the gut microbiota plays an important role in maintaining gastrointestinal homeostasis by converting host nutrients into metabolites. The normal function of the esophagus is maintained through various mechanisms, such as energy metabolism, mucosal barrier repair and immune regulation (Zmora et al., 2019; Gill et al., 2022; Jia et al., 2022). Therefore, the disruption of metabolite balance caused by gut microbiota imbalance may promote GERD.

However, little is known about the interactions between the gut microbiota and metabolites and how they affect the occurrence and development of GERD. In this study, 16S rRNA and metagenomic sequencing were used to assess the characteristics of the gut microbiota in children with GERD, and LC/MS was used to measure serum metabolite levels, providing a new perspective for further revealing the role of the gut microbiota in the pathogenesis of GERD.

## 2 Materials and methods

### 2.1 Subject selection

A total of 30 children with GERD and 30 healthy controls (HCs) who were admitted to Beijing Children’s Hospital Affiliated with Capital Medical University from September 2022 to March 2023 were included in this study. The subjects ranged from 3 to 14 years old. The diagnosis of GERD was made using the relevant criteria in the “Pediatric Gastroesophageal Reflux Disease Diagnosis and Treatment Plan (Trial)” (Wang and Jiang, 2006), and the diagnosis of GERD was confirmed by endoscopy or esophageal pH examination over 24 h. HC children were recruited from child health centers. The exclusion criteria were as follows: (1) use of proton pump inhibitors and gastrointestinal mototropic drugs during the week prior to the study; (2) abnormal anatomical structure of the digestive tract; (3) eosinophilic esophagitis; (4) Helicobacter pylori infection; (5) metabolic diseases; (6) coagulation dysfunction; (7) heart, liver, kidney and other organ functional diseases; and (8) blood and immune system diseases. General information (age, sex, body mass index (BMI)), gastrointestinal symptoms, and routine blood test results were collected from the study subjects, and gastroscopy results and 24-hour esophageal pH test results were also collected from patients with GERD. None of the participants had taken antibiotics, probiotics, or prebiotics in the 2 months prior to stool collection. Thirty children with GERD agreed to provide stool samples collected for 16S rRNA sequencing analysis, 28 children agreed to provide serum samples collected for metabolomics detection analysis, 20 children in the control group provided stool samples collected for 16S rRNA sequencing analysis, and 28 children agreed to provide serum samples used for metabolomics detection analysis (see recruitment Flow Chart 1 for details). This study complied with the principles set out in the Declaration of Helsinki. The study protocol was approved by the Ethics Committee of Beijing Children’s Hospital, and all subjects signed the informed consent form (Approval no. [2022]-E-175-Y).

### 2.2 Fecal sample collection

According to the sample collection instructions, feces were collected in a hospital or at home, transported to Beijing Children’s Hospital affiliated with Capital Medical University under low temperature storage, and then stored in a -80°C refrigerator.

### 2.3 16S rRNA gene sequencing

Total genomic DNA from samples was extracted using the CTAB method (Yang et al., 2022), and the DNA purity and concentration were measured by agarose gel electrophoresis. Appropriate amounts of sample DNA were diluted to 1 ng/μl in a centrifuge tube, and the diluted genomic DNA was used as a template. Using specific primers with barcodes 341 F (5’- TCGTCGGCAGCGTCAGATGTGTATAAGAGACAGCCTACGGGNGGCWGCAG - 3’) and 806 r (5’- GTCTCGTGGGCTCGGAGATGTATATAAGAG ACAGGGACTACHVGGGTWTCTAAT-3’) for 16S bacterial amplicon sequencing (V3-V4 region), the TruSeq^®^ DNA PCR-Free Sample Preparation Kit was used to construct the libraries. After qualified quantitative analysis by Qubit and Q-PCR, NovaSeq 6000 was used for on-machine sequencing. The reads of each sample were spliced by FLASH, and the resulting splicing sequence was the original tag data. The tag sequences were compared with the species annotation database to detect the chimera sequences, and the final valid data were obtained. The Uparse algorithm was used to cluster all valid data obtained from all samples, and sequences with ≥97% similarity were classified as operational taxonomic units (OTUs). The OTU sequence was annotated by species in the SSUrRNA database.

### 2.4 Metagenomic sequencing

After the DNA samples were assessed for quality, libraries were constructed using the NEBNext^®^ Ultra DNA Library Prep Kit for Illumina (NEB, USA). After the DNA samples were assessed for quality, Illumina PE150 sequencing was performed (Treiber et al., 2020). Readfq was used to process the raw data to obtain clean data for subsequent analysis, and the data were assembled and analyzed by MEGAHIT software (v1.0.4-beta). MetaGeneMark was used for gene prediction, CD-HIT software was used to delete redundant genes, DIAMOND software was used for sequence comparison, and MEGAN software’s LCA algorithm was used to determine the species annotation information of the sequences.

### 2.5 Metabolomics analysis

Nontargeted metabolomics analysis was performed by LC/MS technology (Kui et al., 2022). Serum samples with volumes of 100 μL were placed in an eppendorf (EP) tube, 400 μL of 80% methanol solution was added, vortex shocking was carried out, the samples were placed in an ice bath for 5 min, and centrifugation was performed at 15,000 × g and 4°C for 20 min. The supernatant was diluted to achieve a 53% methanol content, and the supernatant was collected for subsequent detection after centrifugation. Ultrahigh-performance liquid chromatography–tandem mass spectrometry (UHPLC–MS/MS) analysis was performed using the Vanquish UHPLC system (Thermo Fisher, Germany) in conjunction with the Orbitrap Q Exactive™ HF Mass spectrometer (Thermo Fisher, Germany). The chromatographic separation conditions were as follows: chromatography was performed on a Hypesil Gold column (C18) at a column temperature of 40°C and a flow rate of 0.2 mL/min. In positive mode, mobile phase A was 0.1% formic acid, and mobile phase B was methanol; in negative mode, mobile phase A was 5 mM ammonium acetate, and mobile phase B was methanol. The detection parameters were as follows: spray voltage, 3.5 kV; sheath gas flow rate, 35 psi; auxiliary gas flow rate, 10 L/min; ion transfer tube temperature, 320°C; ion import RF level, 60; auxiliary gas heater temperature, 350°C; polarity, positive ion and negative ion mode; and MS/MS secondary scanning, data-dependent scanning. The original data were imported into the CD 3.1 library search software for processing, with a mass deviation of 5 ppm, signal strength deviation of 30%, signal-to-noise ratio of 3, and minimum signal strength; ion and other information were used for peak extraction, and quantification of the peak area and integration of the target ion were performed. Then, the molecular formula was predicted using the molecular ion peaks and fragment ions, which were compared with the mzCloud, mzVault and Masslist databases. Finally, the original quantitative results were standardized to obtain the identities and relative levels of metabolites.

### 2.6 Bioinformatics and statistical analysis

SPSS 23.0 was used for statistical analyses of all demographic and clinical data. The quantitative demographic and clinical data with a normal distribution are expressed as the mean ± standard deviation, and the differences between groups were tested by t tests. Quantitative demographic and clinical characteristics with nonnormal distributions are expressed as the medians (P25, P75), and differences between groups were tested using the Wilcoxon rank sum test. Qualitative demographic and clinical data are expressed as percentages. The chi-square test was used to test the differences between groups. The results with p<0.05 were considered statistically significant.

Quantitative Insights Into Microbial Ecology (QIIME) software (Version 1.9.1) was used to calculate the Shannon index, Simpson index and Wilcox test for intergroup difference analysis. Based on the unweighted UniFrac β diversity analysis, principal coordinates analysis (PCoA) visualizations were analyzed using the ggplot2 software package R (Version 2.15.3), and the differences at the phylum, genus and species levels and the functional module abundance between the two groups were analyzed using the Wilcoxon rank sum test. The p value was corrected by the Benjamini–Hochberg method to obtain the q value to control the error rate of multiple tests. Linear discriminant analysis effect size (LEfSe) software was used for LEfSe analysis. DIAMOND software was used to compare the nonredundant gene set with the Kyoto Encyclopedia of Genes and Genomes (KEGG), eggNOG and CAZy databases to obtain the corresponding functional information of gene sequences.

Principal component analysis (PCA) and partial least squares discriminant analysis (PLS-DA) were performed after the metabolite data were transformed by MetaX software. The analysis of intergroup metabolite differences was performed using a t test, and the fold change (FC) between groups was calculated. The default criteria for differential metabolite screening were variable importance of projection (VIP)>1, P <0.05, FC≥2 or FC ≤ 0.5. The ggplot2 package of R software was used to draw the volcano map, and the Pheatmap package was used to draw the clustering heatmap using the z score to normalize the metabolite data. The identified metabolites were annotated by the KEGG, HMDB and LIPID Maps databases. The KEGG metabolic pathways enriched by differential metabolites were identified, and results with P <0.05 were considered to indicate significant enrichment. Spearman correlation coefficients were calculated using the levels of the differentially enriched bacteria and metabolites in R software.

## 3 Results

### 3.1 Alterations in the gut microbiota composition in children with GERD based on 16S rRNA sequencing

**Figure 1:**
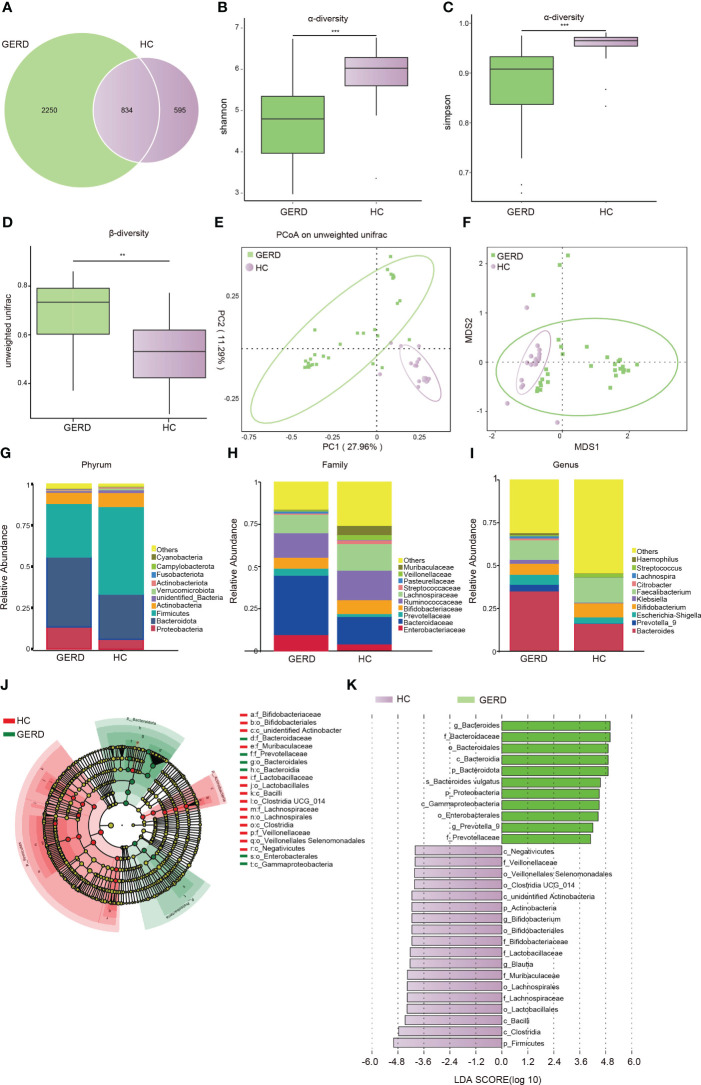
Changes in the gut microbiota based on 16S rRNA data **(A)** Venn diagram; **(B)** α-Diversity based on the Shannon index between the groups; **(C)** α-diversity based on the Simpson index between the groups; **(D)** β-diversity based on the unweighted UniFrac distance; **(E)** PCoA based on the unweighted UniFrac distance; **(F)** NMDS analysis; **(G)** microbiota composition at the Phylum level; **(H)** microbiota composition at the family level; **(I)** microbiota composition at the genus level. **(J, K)** LEfSe analysis showing the phylogenetic differences between the groups. Only results with LDA > 4 are shown. **p < 0.01, ***p <0.001.

There were no statistically significant differences in age, sex, or BMI among all participants. Details of the demographic and clinical characteristics of the children with GERD and HCs are shown in **Supplementary Table S1**. To determine whether the gut microbiota composition of children with GERD was changed as compared to HC, 16S rDNA sequencing was used to detect fecal microbes in children with GERD and HC children. The species Venn diagram showed a total of 834 OTUs in the two groups, with 2250 OTUs specific to the GERD group and 595 OTUs specific to the control group (**Figure 1A**). The α diversity results showed that the Shannon and Simpson indices in children with GERD were significantly lower than those in the HC group, indicating a decrease in gut microbiota diversity in children with GERD (**Figures 1B, C**). Analysis of similarities (ANOSIM) based on unweighted UniFrac distance showed significant differences in the distribution of the microbial community structure between the two groups (**Figure 1D**). PCoA and nonmetric multidimensional scaling (NMDS) showed a difference in the gut microbiota composition between the two groups (**Figures 1E, F**). At the phylum level, the relative abundances of Proteobacteria and Bacteroidetes in the GERD group were significantly higher than those in the HC group, while the relative abundances of Firmicutes and Actinobacteria were significantly lower (**Figure 1G**). At the family level, the relative abundances of *Enterobacteriaceae*, *Bacteroidaceae* and *Prevotellaceae* in children with GERD were high, and those of *Bifidobacteriaceae*, *Ruminococcaceae*, and *Lachnospiraceae* were low (**Figure 1H**). At the genus level, the relative abundances of *Bacteroides*, *Prevotella-9*, *Escherichia-Shigella*, *Klebsiella* and *Haemophilus* in children with GERD were significantly higher than those in the HC group. The relative abundances of *Bifidobacterium*, *Faecalibacterium* and *Streptococcus* were lower in the GERD group than in the HC group (**Figure 1I**). LEfSe analysis was used to process the samples to identify species with significant differences between the groups. The results showed that *Prevotella-9* and *Bacteroides* had the highest abundances in the GERD group, while *Bifidobacterium* and *Blautia* had the highest abundances in the HC group (**Figures 1J, K**).

### 3.2 Metagenomic sequencing showed significant differences in the gut microbiota composition between children with GERD and HC children

**Figure 2:**
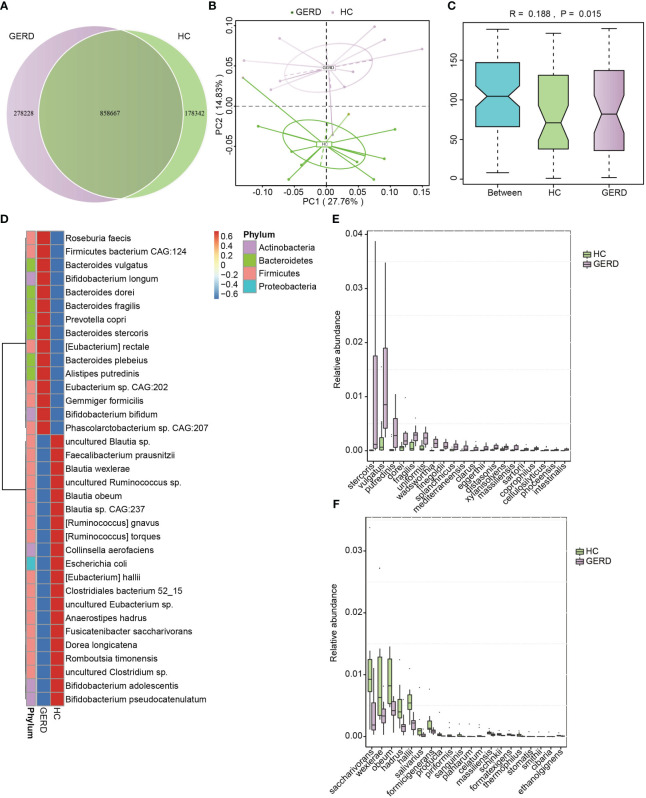
Gut microbiota differences based on metagenomic sequencing. **(A)** Venn diagram; **(B)** PCoA based on the Bray–Curtis distance; **(C)** ANOSIM analysis based on species abundance, Between represents the difference between groups; others are within groups; **(D)** Clustering heatmaps of the differentially enriched bacterial species between the groups; **(E, F)** The top 20 microbes with significant differences in abundance at the species level.

To determine the changes in the gut microbiota at the species level in children with GERD, metagenomic sequencing was performed using stool samples from 10 children with GERD and 10 age-matched HC children after sample selection through microPITA. The Venn diagram results showed that the two groups had a total of 858,667 genes, with 278,228 specific genes in the GERD group and 178,342 specific genes in the HC group (**Figure 2A**). The PCoA results based on the Bray–Curtis distance showed significant separation of the microbial composition at the species level between the GERD and HC groups (**Figure 2B**), and ANOSIM confirmed the significant differences in the gut microbiota composition at the species level between the two groups (**Figure 2C**). The clustering heatmap of bacterial species enriched by differences between the GERD and HC groups is shown in **Figure 2D**. **Figures 2E, F** show the top 20 species of bacteria that were significantly enriched in the gut microbiota of the GERD group and HC group, respectively. The abundances of *Bacteroides stercoris*, *Bacteroides vulgatus*, *Alistipes putredinis*, *Bacteroides dorei*, and *Bacteroides fragilis* increased significantly, while those of *Fusicatenibacter saccharivorans*, *Blautia wexlerae*, *Blautia obeum*, *Anaerostipes hadrus* and *Eubacterium hallii* decreased significantly.

### 3.3 Functional changes in the gut microbiota in children with GERD

**Figure 3:**
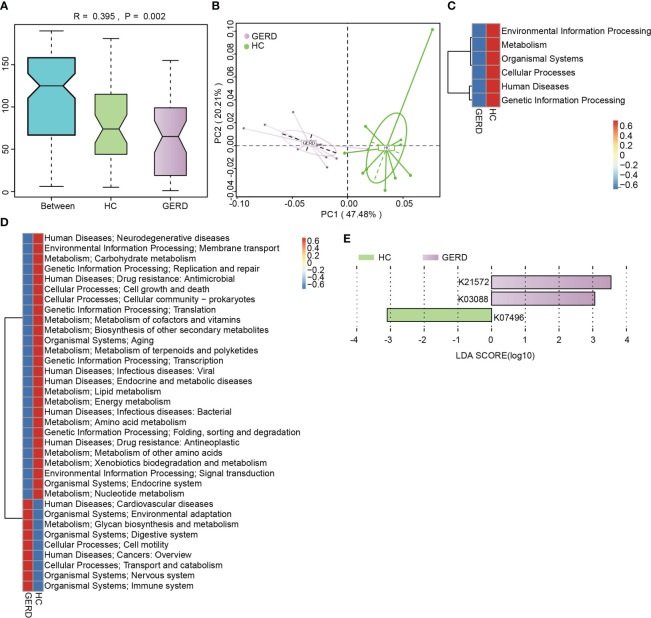
KEGG functional annotation of the gut microbiota genome. **(A)** ANOSIM analysis based on KO levels, Between represents the difference between groups; others are within groups; **(B)** PCoA on the Bray–Curtis distance; **(C)** Level 1 clustering heatmap of the KEGG modules; **(D)** Level 2 clustering heatmap of the KEGG modules; **(E)** Histogram of the LEfSe analysis based on the KO level (LDA>3).

To identify the functional characteristics of the gut microbiota of children with GERD, this study further annotated the gut microbiota metagenomic data using the KEGG database, and ANOSIM indicated that the gut microbiota differed significantly between the GERD group and the HC group at the KEGG Orthological (KO) level (**Figure 3A**). The PCoA results confirmed that there were significant differences in the distribution of microbial functions based on the KEGG module between the GERD and HC groups (**Figure 3B**). The activities of metabolic pathways such as energy, amino acids, vitamins, carbohydrates and lipids were lower in the GERD group than in the HC group, while the activities of glycan synthesis and metabolic pathways were higher (**Figures 3C–E**).

The eggNOG orthologous group (og) in the GERD group was also significantly separated from that in the HC group (**Supplementary Figure S1A**). The PCoA results showed significant differences in the distribution of functional genes between the two groups (**Supplementary Figure S1B**). The functions of cell cycle control, cell division, cytoskeleton, extracellular structure, nucleotide transport and metabolism, energy production and conversion, etc., were more impaired in the GERD group than in the HC group, while cell motility, cell wall/membrane/envelope biogenesis, inorganic ion transport and metabolic pathway enrichment were affected (**Supplementary Figures S1C–E**).

### 3.4 Abnormal metabolic patterns in children with GERD

**Figure 4:**
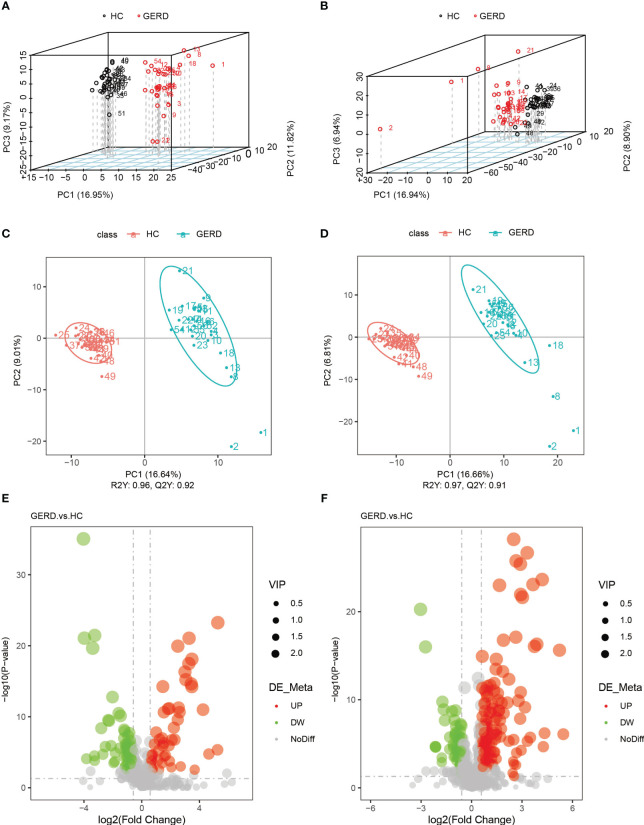
Serum metabolomics analysis of the groups. **(A, B)** PCA based on metabolites of serum samples in negative ion mode **(A)** and positive ion mode **(B)**; **(C, D)** PLS-DA based on serum sample metabolites in negative ion mode **(C)** and positive ion mode **(D)**; **(E, F)** Volcano maps of differential metabolites under negative ion mode **(E)** and positive ion mode **(F)**.

The above metagenome-based functional annotation analysis revealed functional changes in the gut microbiota in children with GERD. In this study, the serum samples of the two groups of children were used for metabolomic analysis by LC/MS technology. Principal component analysis (PCA) and PLS-DA of serum metabolites were performed. The PLS-DA results showed significant differences in the metabolic characteristics of serum samples between the GERD group and the HC group (**Figures 4A–D**). The PLS-DA model verified that there was no overfitting phenomenon, and the sample characteristics could be used for subsequent analysis (**Supplementary Figure S2**). Furthermore, the differential metabolites between the two groups were analyzed by volcano plot, and the threshold values were set as VIP > 1.0, FC > 1.5 or FC < 0.667 and P value< 0.05. The results showed that 182 differential metabolites were identified in the positive ion mode between the GERD and HC groups, of which 130 metabolites had increased levels in the GERD group. The levels of 52 metabolites were lower in the GERD group. In negative ion mode, 106 differential metabolites were identified between the groups, of which 49 had higher levels and 57 had lower levels in the GERD group (**Figures 4E, F**).

**Figure 5:**
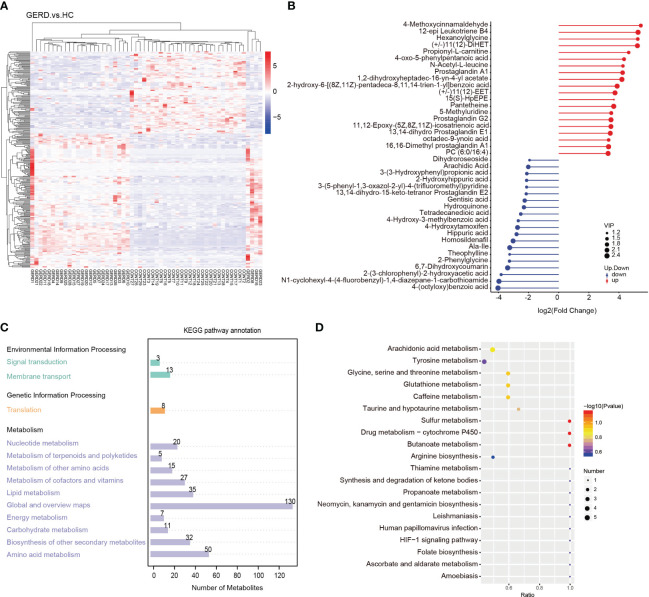
Aberrant metabolic patterns in GERD patients **(A)** Differential metabolite cluster heatmaps; **(B)** Matchstick map of the differentially abundant metabolites; **(C)** Differentially abundant metabolite KEGG pathway annotation diagram; **(D)** Bubble map of the KEGG enrichment analysis.

The results of the clustering heatmap showed that samples obtained from the same tissue were clustered into a group, and the relative content of metabolites in different sample groups significantly differed (**Figure 5A**). According to the differential multiple value of the metabolites in the two groups, logarithmic conversion was performed with the base of 2, and the top 20 metabolites in this dataset were selected and displayed in the matchstick chart. As shown in **Figure 5B**, the levels of metabolites such as 4-methoxycinnamaldehyde, 12-epi leukotriene B4, propionyl-L-carnitine, prostaglandin A1, and prostaglandin G2 in the GERD group were significantly increased. The levels of metabolites such as 4-benzoic acid, gentisic acid, hydroquinone, tetradecanediacid, 2-phenylglycine and theophylline decreased significantly. Further KEGG annotation of the differential metabolites showed that the top three enrichment pathways were global and overview maps, amino acid metabolism, and lipid metabolism (**Figure 5C**). The KEGG bubble map shows the top 20 pathways with the most significant enrichment. The metabolic pathways affected by the differences in metabolites between children with GERD and HCs mainly included arachidonic acid (AA) metabolism; tyrosine metabolism; sulfur metabolism; glycine, serine, and threonine metabolism; glutathione metabolism; and caffeine metabolism (**Figure 5D**).

### 3.5 Correlation analysis between the gut microbe and metabolite levels

**Figure 6:**
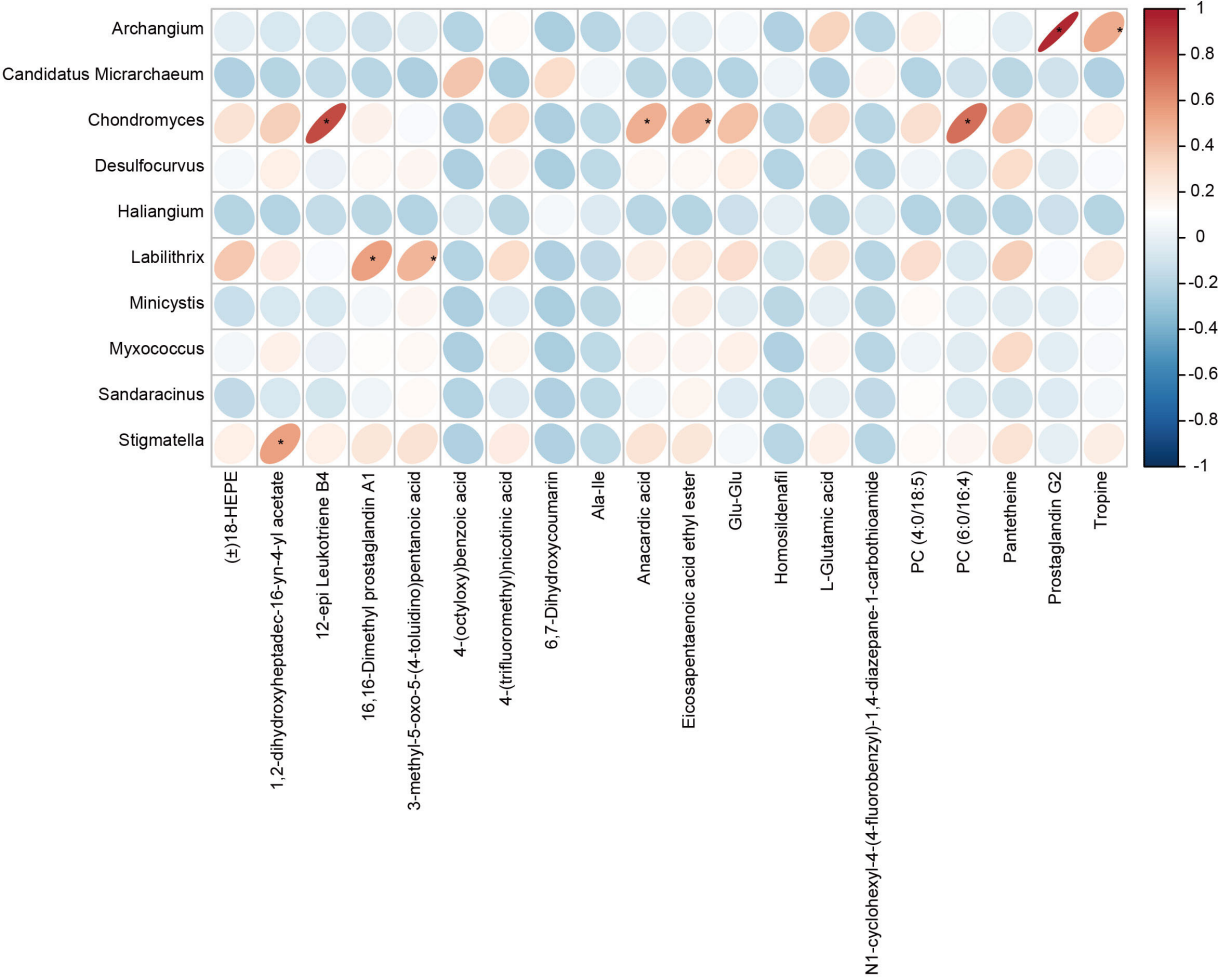
Correlation heatmap between the fecal microbiota and serum metabolites The rows represent differentially abundant microbes, the columns represent differentially abundant metabolites, and the legend on the right provides the correlation coefficient, with red indicating a positive correlation and blue indicating a negative correlation, *P < 0.05.

Pearson correlation analysis was used to analyze the associations between the differential gut microbes in children with GERD and serum metabolites, and the results showed that the changes in serum metabolites in children with GERD were significantly correlated with the changes in the levels of various bacteria in the gut microbiota. The levels of *Chondromyces*, *Labilithrix* and *Stigmatella* were significantly positively correlated with the levels of a variety of metabolites (**Figure 6**), suggesting significant interactions between the gut microbiota and metabolites that may affect the occurrence of GERD.

## 4 Discussion

The imbalance in the gut microbiota and its related metabolites may lead to the occurrence and development of GERD. However, recent studies on the microbiota in individuals with GERD are based on 16S rRNA sequencing and mainly focus on esophageal microorganisms, and information on the role of the gut microbiota in the pathogenesis of GERD is still limited. Therefore, it is necessary to conduct in-depth classification and functional identification of the gut microbiota in individuals with GERD. Using metagenomic and metabolomic analysis, this study confirmed that gut microbiota dysregulation exists in children with GERD, and specific intestinal bacterial genera and their related functional transformations are closely related to the circulating metabolites associated with GERD, revealing the potential host-intestinal microbiota-metabolite interaction involved in the pathogenesis of GERD.

The rich gut microbiota and its structural diversity help the host maintain a balance in metabolite levels and energy metabolism and effectively resist invasion by pathogenic microorganisms (Nicolas and Chang, 2019; Takeuchi and Ohno, 2021; Gill et al., 2022). Decreased gut microbiota diversity is associated with obesity, allergic diseases and functional gastrointestinal disorders (Wu et al., 2021; Wu et al., 2021; Akagawa and Kaneko, 2022; Akagawa and Kaneko, 2022; Pan et al., 2022; Pan et al., 2022; Zhang et al., 2022; Puljiz et al., 2023; Puljiz et al., 2023). Previous studies have shown reduced esophageal microbial diversity in children with BE (Elliott et al., 2017). Similar to children with eosinophilic esophagitis (EoE) (Kashyap et al., 2019), the gut microbiota structure of children with GERD showed significant changes, with a decreased α diversity index and an altered β diversity index. The Shannon and Simpson indices of children with GERD were significantly reduced, and the PCoA and NMDS results also confirmed the differences in the gut microbiota distribution and composition between children with GERD and HCs. Taken together, these results suggest that both the α diversity and β diversity indices provide strong evidence for gut microbiota dysregulation in children with GERD.

The classification of bacteria at the phylum, family, genus and species levels in children with GERD significantly differed from that in the HC group. The results of this study showed that Proteobacteria and Bacteroidetes levels increased significantly in children with GERD, and Firmicutes and Actinobacteria levels decreased significantly. The distribution characteristics of this increase in the levels of Proteobacteria and decrease in the levels of Firmicutes and Actinobacteria are similar to the distribution of the gut microbiota in children with acute radiation esophagitis (Lin et al., 2022). At the family level, *Enterobacteriaceae* levels were significantly increased in children with GERD. Studies have shown that *Enterobacteriaceae* is related to a variety of esophageal diseases, such as esophagitis and BE, and may mediate esophageal mucosal inflammation and intestinal metaplasia (Amir et al., 2014). In addition, *Blautia* and *Lachnospira* in *Lachnospiraceae* and *Faecalibacterium* in *Ruminococcaceae* are associated with the production of SCFAs. SCFAs are produced by the fermentation of indigestible dietary fiber by bacteria in the gut. SCFAs mainly include acetate, propionate and butyrate. In addition to being the main energy source used by colon cells, SCFAs function in the bidirectional regulation of colonic motility, maintenance of intestinal homeostasis and improvement in the intestinal barrier (Correa-Oliveira et al., 2016; Koh et al., 2016; Parada Venegas et al., 2019; Yue et al., 2022; Tan et al., 2023). Although SCFAs are primarily produced in the gut, they have been shown to have immunomodulatory effects on other barrier organs (Smolinska et al., 2017; Krejner et al., 2018). Butyrate and propionate SCFAs are effective in improving esophageal epithelial barrier function (Kleuskens et al., 2022). Butyrate regulates host inflammation through G protein-coupled receptors and inflammatory pathways such as the NF-κB pathway (Bach Knudsen et al., 2018; Huang et al., 2020; Lu et al., 2022). In addition, SCFAs can affect the occurrence of parenteral immune and inflammatory responses after entering the circulation through active transport mediated by monocarboxylate transporters (Borthakur et al., 2008; Soret et al., 2010; Zhan et al., 2018). Given the above findings that reduced SCFA levels in the gut microbiota of children with GERD may mediate the development of esophageal inflammation, more studies are needed to evaluate the therapeutic potential of butyrate and other SCFAs in GERD. Moreover, previous studies have found that LPS relaxes the lower esophageal sphincter and delays gastric emptying by inducing the production of nitric oxide synthase and cyclooxygenase-2 (Yang et al., 2012). Consistent with this information, this study further revealed a significant increase in the levels of the gut microbes *Haemophilus* and *Klebsiella*, which are involved in LPS synthesis in children with GERD, and *Klebsiella* is involved in LPS-mediated NF-κB activation and the production of inflammatory factors, such as TNF-α, IL-6 and IL-1β, through TLR4 (Sa-Pessoa et al., 2020). In conclusion, this study not only provides direct evidence for gut microbiota disturbance in children with GERD but also provides an in-depth understanding of the relationship between gut microbiota disturbance and specific functional changes as well as the possible mechanism of its involvement in the pathophysiological process of GERD.

Metabolomics is a branch of omics technology that detects the metabolites of all small-molecule compounds in an organism in a high-throughput manner. Metabolomic analysis of biological samples such as serum or urine has been widely used in clinical practice. Biomarker screening is used for disease diagnosis, activity monitoring, prognostic analysis, and efficacy prediction (Johnson et al., 2016; Khamis et al., 2017; Rinschen et al., 2019). In this study, LC/MS technology was used to identify all the different metabolites in the serum of children with GERD and HC children. Multivariate analysis was performed to identify the differences between the two groups, and 288 different metabolites were screened. Among the pathways involving these metabolites, the top 5 differential metabolic pathways were AA metabolism; tyrosine metabolism; glycine, serine and threonine metabolism; glutathione metabolism; and caffeine metabolism. Notably, the AA metabolic pathway is mainly used to synthesize inflammatory mediators, which can mediate the production of various inflammatory factors, such as TNF-α, IL-1β, IFN-γ, and MCP-1. Thus, the AA metabolic pathway is closely related to the occurrence and development of inflammation (Zhuang et al., 2017; Gonzalez-Perilli et al., 2019; Hellstrom et al., 2020). The results of this study showed significant changes in serum metabolites related to AA metabolism in children with GERD, suggesting that the occurrence and development of GERD may be related to abnormal AA metabolism. A recent study showed that neuAcalpha2-3Galbeta-Cer (d18:1/16:0), sphinganine, and AA can be used as serum diagnostic biomarkers in children with GERD (AUC: 0.842, 95% CI 0.704-0.98) (Ye et al., 2021). *In vivo*, AA is converted to 5-hydroperoxy-eicosatetraenoic acid by binding to the 5-lipoxygenase activating protein, which is later oxidized to leukotriene A4 (LTA4). After formation, it is converted into the inflammatory cell chemokine leukotriene B4 (LTB4) by LTA4 hydrolase, making the latter a potential new target for the treatment of inflammatory diseases (Bouchareychas et al., 2017; Jala et al., 2017; Saeki and Yokomizo, 2017). Studies have shown that the level of LTB4 in the esophageal mucosa of children with RE and BE is significantly higher than that in controls (Triadafilopoulos et al., 1996). In this study, LTB4 levels were significantly higher in children with GERD than in HCs, suggesting that LTB4 may serve as a novel biomarker for the diagnosis of GERD.

Comprehensive analysis of diseased microbiomes and nontarget metabolomes initially revealed the relationship between differential microorganisms and differential metabolites and pointed to AA metabolism, the main lipid metabolic pathway. Our multiomics studies demonstrate correlations between different bacterial genera and metabolites, and the reason for these differentially expressed metabolites may be alterations in the structure of the gut microbiota. We hypothesize that the colonization of the digestive tract by harmful bacteria such as *Haemophilus*, *Chondromyces*, and *Klebsiella* during the pathogenesis of GERD and the decrease in colonization of beneficial bacteria such as *Blautia*, *Lachnospira* and *Faecalibacterium* affect the host immune system through host-microbiota interactions, affect metabolic pathways such as AA and promote the esophageal inflammatory response. There is growing evidence that bacterial metabolites, toxins, and structural components from pathogenic and opportunistic bacteria may stimulate harmful immune responses that can lead to the onset of esophageal disease(Verbeek et al., 2014; Peng et al., 2023).

There are still some limitations to this study. First, this study was a cross-sectional study, which illustrated the high correlation between the gut microbiota and metabolites and the occurrence of GERD but could not establish a causal relationship. In the future, this relationship can be further verified through animal models in which the gut microbiota mediates esophageal inflammation by affecting AA metabolism to clarify the causal relationship and study the specific pathophysiological mechanisms involved and possible intervention measures. In addition, because our cohort sample size was small and included only Chinese individuals, further research is needed to determine whether the conclusions of this study apply to populations with other ethnicities.

## 5 Conclusions

In conclusion, this study not only revealed the imbalance in the gut microbiota in children with GERD but also revealed the imbalance and functional changes among specific core bacteria in the gut microbiota of children with GERD and further analyzed the relationship between the gut microbiota and metabolite changes. These results not only provide direct evidence for the hypothesis that a GERD-associated gut microbiota imbalance exists but may also provide strong evidence for further studies of the pathogenesis of GERD.

## Data availability statement

The datasets presented in this study can be found in online repositories. The names of the repository/repositories and accession number(s) can be found below: https://www.ncbi.nlm.nih.gov/, PRJNA993632 and PRJNA996590.

## Ethics statement

The studies involving humans were approved by Medical Ethics Committee of Beijing Children’s Hospital Affiliated to Capital Medical University. The studies were conducted in accordance with the local legislation and institutional requirements. Written informed consent for participation in this study was provided by the participants’ legal guardians/next of kin. Written informed consent was obtained from the individual(s), and minor(s)’ legal guardian/next of kin, for the publication of any potentially identifiable images or data included in this article.

## Author contributions

XY: Writing – original draft, Writing – review & editing, Data curation, Investigation, Visualization. FY: Conceptualization, Data curation, Methodology, Writing – original draft. JZ: Data curation, Formal Analysis, Methodology, Writing – original draft. CZ: Investigation, Resources, Validation, Writing – original draft. JW: Writing – review & editing, Funding acquisition, Resources. XN: Writing – review & editing.
