# Supplementary figures and images for: Analysis of the gut microbiota in children with gastroesophageal reflux disease using metagenomics and metabolomics
Source: Front Cell Infect Microbiol. 2023 Oct 13;13:1267192. doi: 10.3389/fcimb.2023.1267192 (PMC10613033; doi:10.3389/fcimb.2023.1267192)

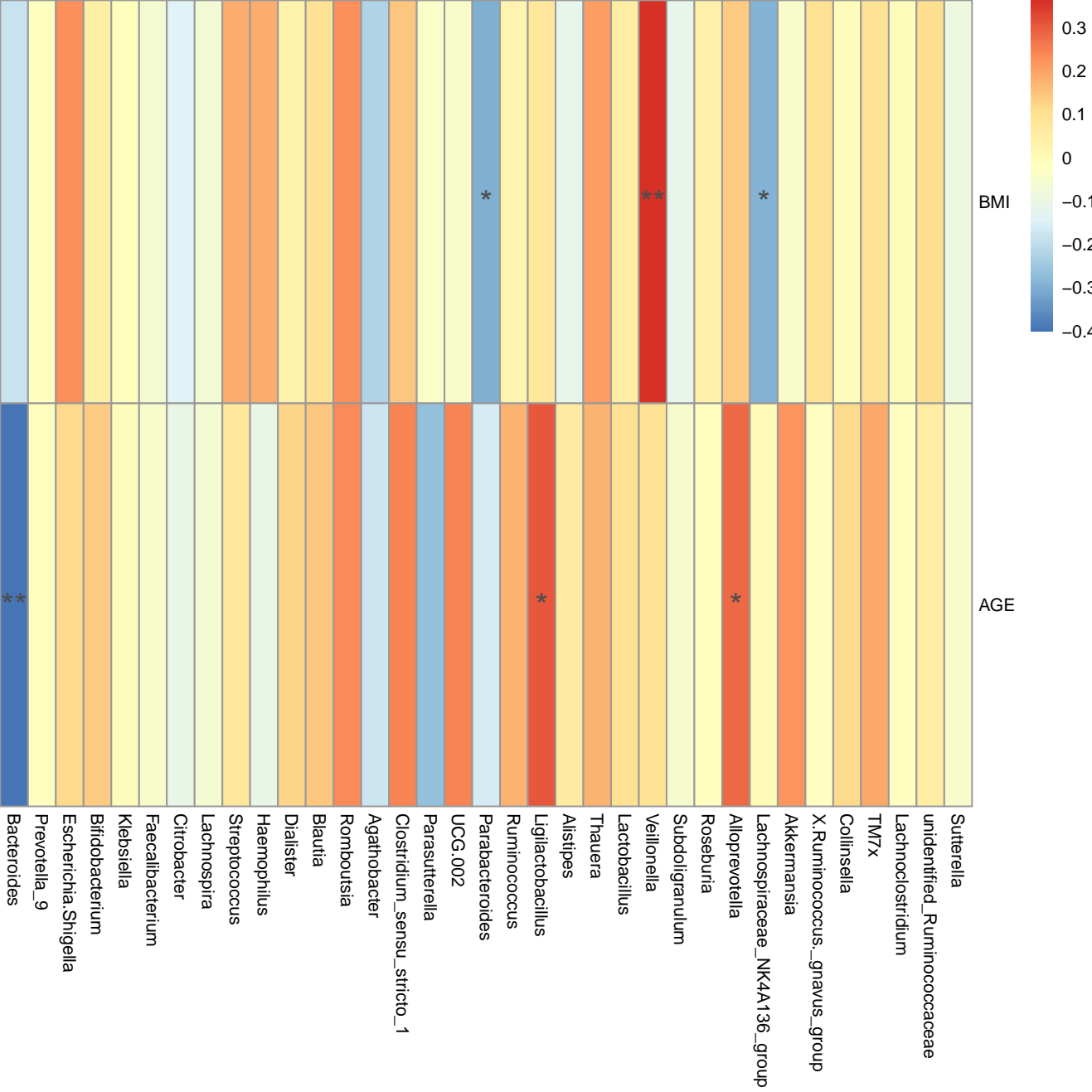

Supplement: Supplementary file 1 [file DataSheet_1.pdf]

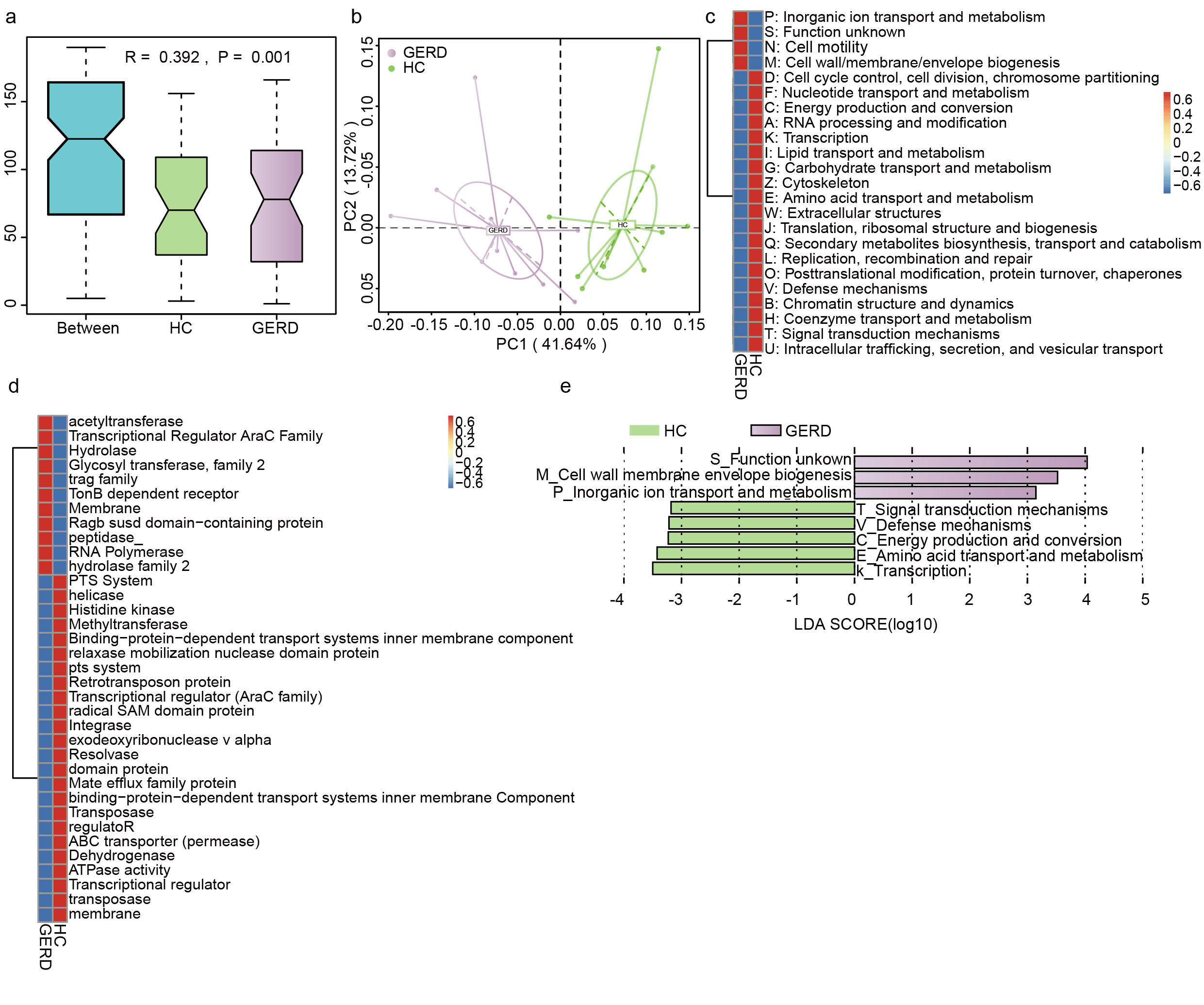

Supplement: Supplementary file 2 [file Image_1.jpeg]

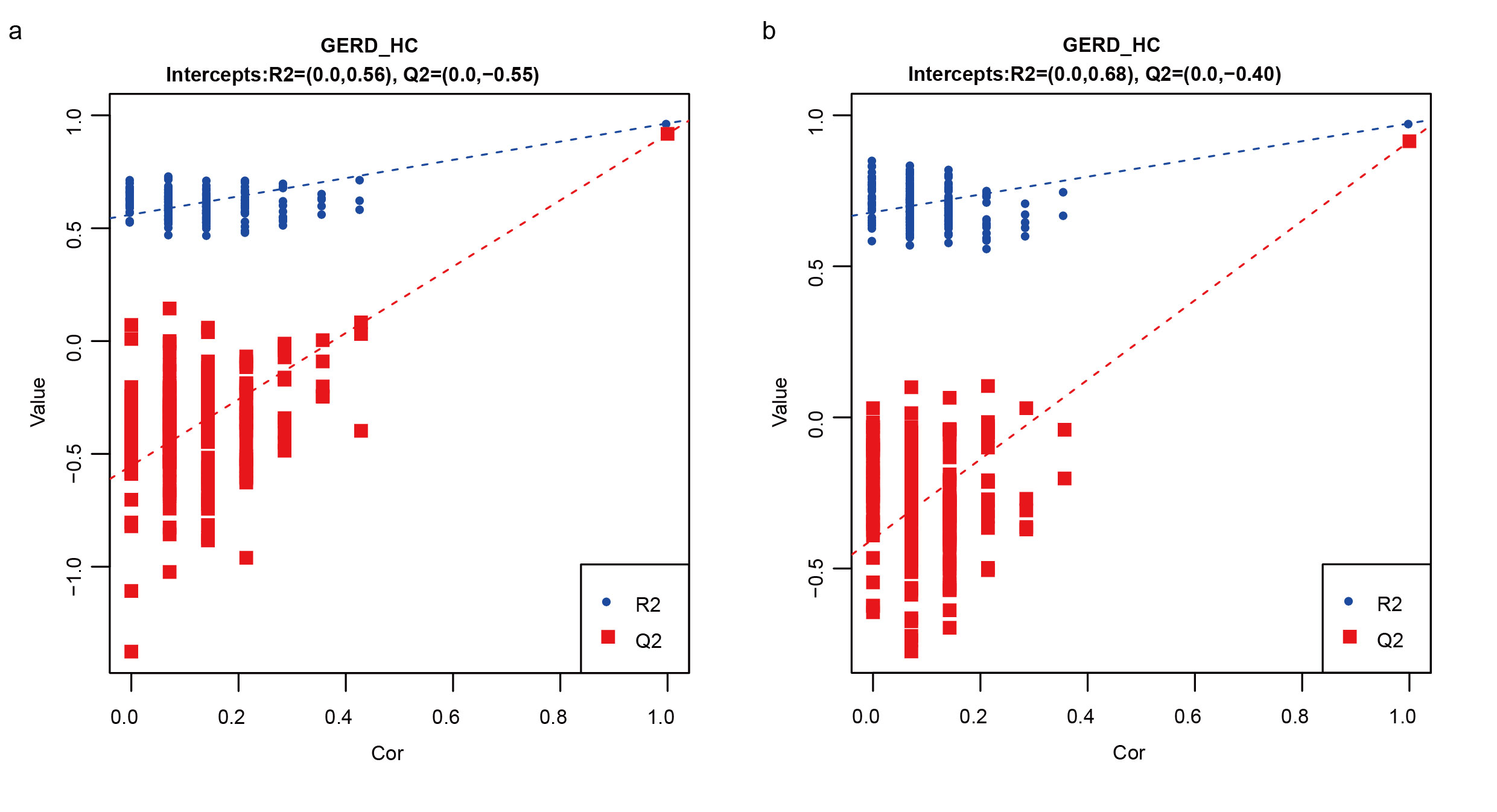

Supplement: Supplementary file 3 [file Image_2.jpeg]
