# Supplementary material for: Analysis of the gut microbiota in children with gastroesophageal reflux disease using metagenomics and metabolomics
Source: Front Cell Infect Microbiol. 2023 Oct 13;13:1267192. doi: 10.3389/fcimb.2023.1267192 (PMC10613033; doi:10.3389/fcimb.2023.1267192)
Supplement: Supplementary file 4 [file Table_1.docx]

**Table S1. Patient characteristics.**

|  | GERD  (n=30) | HC  (n=30) | p value |
| --- | --- | --- | --- |
| Sex, male, n (%) | 19 (63.3) | 20 (66.7) | 0.787^b^ |
| Age (years), median ±SD | 9.07±3.151 | 9.9±2.67 | 0.274^a^ |
| BMI (kg/m2), median ±SD | 17.401±3.7062 | 17.792±1.6306 | 0.599^a^ |
| **Main symptom reported, n (%)** |  |  |  |
| Heartburn or chest pain | 24 (80) |  |  |
| Regurgitation | 28 (93.3) |  |  |
| Vomiting | 21 (70) |  |  |
| Respiratory symptoms | 9 (30) |  |  |

a = Student’s t test b = chi-square test

Abbreviations: SD, standard deviation; BMI, body mass index.
